# Clinical utility of metagenomic next-generation sequencing in the diagnosis of severe influenza complicated by invasive pulmonary aspergillosis

**DOI:** 10.3389/fcimb.2026.1746504

**Published:** 2026-04-21

**Authors:** Siqiang Niu, Limin Guo, Zhihai Li, Yanshan Liu, Limin Zhao

**Affiliations:** 1 Xinxiang Central Hospital, Xinxiang, Henan, China; 2 Xinxiang Key Laboratory of Respiratory Diseases, Xinxiang, Henan, China; 3 Department of Respiratory and Critical Care Medicine, Henan Provincial People’s Hospital, Zhengzhou, Henan, China; 4 Zhengzhou University People’s Hospital, Zhengzhou, Henan, China; 5 People’s Hospital of Henan University, Zhengzhou, Henan, China

**Keywords:** diagnosis, GM test, influenza, invasive pulmonary aspergillosis, metagenomic next-generation sequencing

## Abstract

**Objective:**

The incidence and mortality of severe influenza complicated by invasive pulmonary aspergillosis (IPA) have risen markedly in recent years. This study aimed to evaluate the diagnostic performance of metagenomic next-generation sequencing (mNGS) for detecting IPA in patients with severe influenza.

**Methods:**

Severe influenza patients with suspected of having IPA admitted to Xinxiang Central Hospital, Henan Province, China, from March 2020 to September 2025 were retrospectively enrolled. Bronchoalveolar lavage fluid (BALF) and blood were collected for fungal culture, galactomannan (GM) assay, and mNGS. Final classification into IPA and non-IPA groups was based on composite clinical and microbiological criteria. Sensitivity, specificity, and receiver operating characteristic curves were used to compare the diagnostic performance of the three methods.

**Results:**

Comparison with traditional fungal culture and GM testing, mNGS provided significantly faster results. Among 189 patients suspected of severe influenza-associated IPA, mNGS demonstrated a sensitivity of 72.1% and a specificity of 80.2%. Its sensitivity was higher than that of fungal culture (28.6%), serum GM testing (37.6%), and BALF GM testing (44.1%); however, its specificity was slightly lower than that of fungal culture (89.5%), serum GM testing (84.3%), and BALF GM testing (81.3%). The area under the ROC curve (AUC) for mNGS was 0.76, which is higher than that for BALF GM testing (0.63), serum GM testing (0.61), and fungal culture (0.59). The combined diagnostic approach yielded an AUC of 0.83.

**Conclusion:**

mNGS offers a rapid, sensitive and accurate solution for invasive pulmonary aspergillosis in severe influenza patients. It outperforms conventional fungal culture and galactomannan assays. Integrating mNGS with traditional diagnostic methods could substantially improve early detection and overall yield of IPA.

## Introduction

Following the influenza pandemic, IPA has increasingly been recognized as a significant and common complication of influenza-associated pneumonia, leading to a marked increase in morbidity and mortality, particularly in critically ill patients. By June 2018, a total of 128 instances of influenza-associated Aspergillus infections had been reported, with a mortality rate estimated at 40%, significantly exceeding the 20% rate observed in influenza patients without IPA (Schwartz et al., 2020).

Although influenza-associated IPA carries a high mortality rate and poor prognosis, making early diagnosis critically important, the clinical and radiological features of influenza-associated IPA are frequently non-specific, which makes microbiological confirmation essential for diagnosis. Traditional diagnostic techniques for Aspergillus infections, such as fungal culture (Schwartz et al., 2020; Verweij et al., 2020),-β-D-glucan (G test), and GM assay, present several limitations, including long processing times and limited sensitivity. These shortcomings frequently lead to delays in both the diagnosis and the initiation of appropriate antifungal treatment.

mNGS represents a promising diagnostic technology that enables comprehensive, unbiased pathogen detection directly from clinical specimens without the need for targeted primers or probes. Although mNGS has been extensively employed across a broad spectrum of infectious diseases for pathogen identification, its diagnostic performance in severe influenza complicated by IPA remains inadequately characterized (Huang et al., 2019). This study aimed to systematically evaluate the diagnostic efficacy of mNGS in patients with severe influenza suspected of IPA. Through comparative analysis with conventional methods, including fungal culture, serum GM testing, and BALF GM testing, the clinical value of mNGS in improving diagnostic accuracy and guiding early antifungal management was comprehensively assessed.

## Materials and methods

### Study design and patients

**Figure 1:**
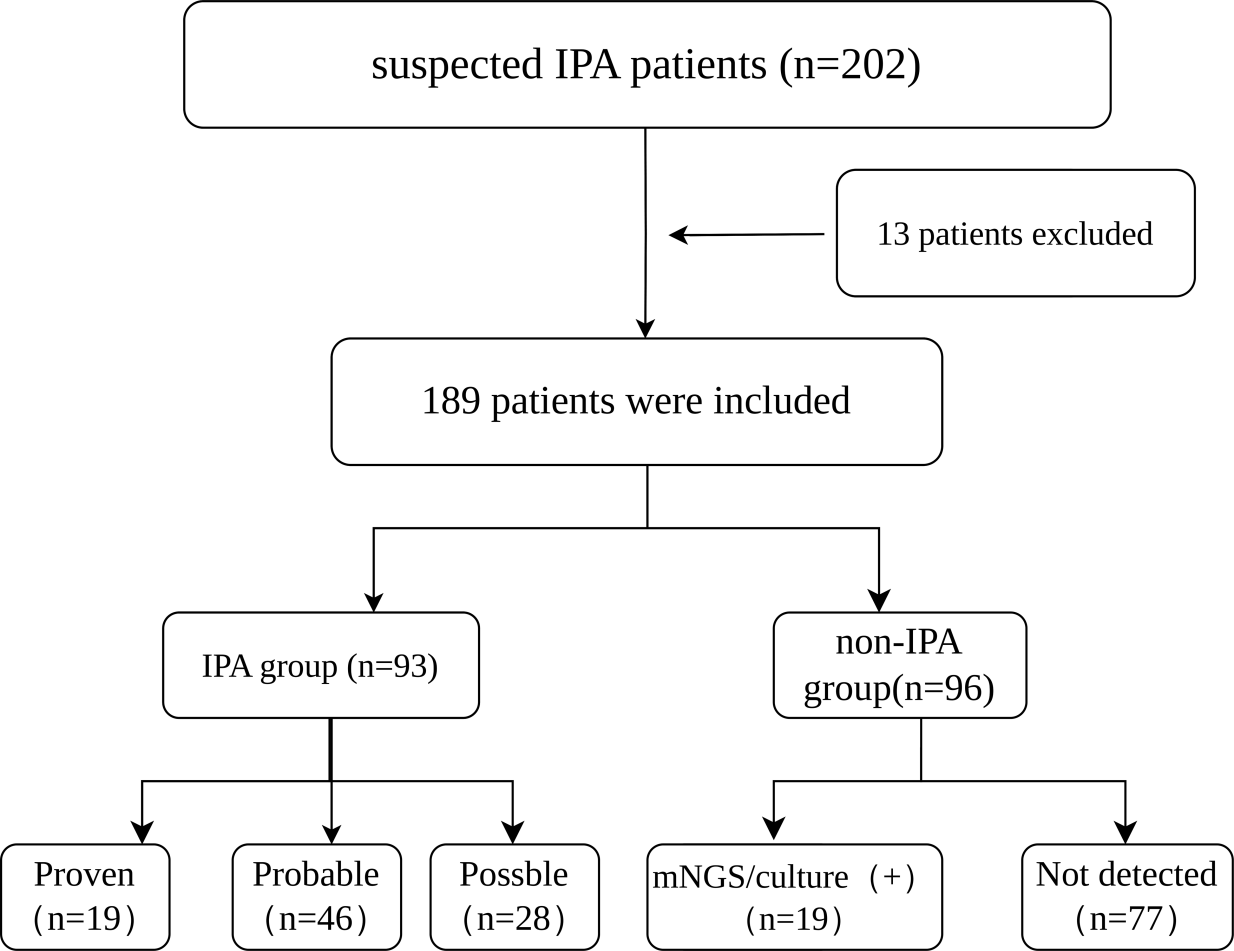
Flowchart of study cohort identification.

This retrospective study was conducted in the Department of Respiratory and Critical Care Medicine at Xinxiang Central Hospital (Henan Province, China) between March 2020 and September 2025. The inclusion criteria were as follows (1): positive influenza PCR or antigen testing, accompanied by respiratory distress (2); presence of clinical features consistent with pulmonary infection, including: a. persistent fever (>96 hours) unresponsive to appropriate antibacterial therapy; b. pulmonary symptoms and signs suggestive of lower respiratory tract infection; c. chest CT findings indicative of possible pulmonary aspergillosis (Verweij et al., 2020); completion of bronchoalveolar lavage (BAL) with available results for fungal culture, GM antigen testing, and mNGS (4); bronchoscopic evidence of airway pseudomembranes, ulcers, nodules, or plaques (Verweij et al., 2020). Patients were excluded if they met any of the following criteria (1): diagnosis of non-severe or non-critical influenza (2); incomplete clinical data or treatment records. Patients fulfilling the inclusion and exclusion criteria were classified as suspected IPA cases. The final diagnosis was determined according to clinical, radiological, and microbiological evidence. A total of 189 patients were enrolled, comprising 93 confirmed IPA cases and 96 non-IPA cases (**Figure 1**).

Ethics approval for this study was obtained from the Ethics Review Board of Xinxiang Central Hospital (Henan Province, China). The need for individual informed consent was formally waived due to the retrospective nature of the research.

### Data collection

Data on patient demographics and clinical course comprised age, sex, comorbidities, laboratory parameters, imaging data, and ICU management specifics (ranging from length of ICU stay and SOFA score to the duration of mechanical ventilation).

### Diagnostic criteria for influenza and severe influenza

The diagnosis of influenza was based on the presence of characteristic clinical symptoms and at least one positive laboratory test result, including real-time polymerase chain reaction (RT-PCR), viral culture, or viral antigen testing.

In addition to meeting the diagnostic criteria for influenza, patients were also required to satisfy the criteria for severe influenza according to the “2025 Influenza Diagnosis and Treatment Guidelines” for critical influenza cases.

### Diagnosis of IPA

Diagnosis of IPA was established using a modified version of the EORTC/MSG criteria. The defining clinical features included new pulmonary infiltrates on chest X-ray or CT scans, accompanied by one or more of the following symptoms: persistent fever, dyspnea, hemoptysis, or respiratory distress. Microbiological evidence consisted of at least one of the following: positive culture for *Aspergillus* species, detection of galactomannan (GM) antigen in serum (≥0.5), or positive GM antigen in bronchoalveolar lavage fluid (BALF, optical density ≥1.0). Based on the EORTC/MSG criteria, patients were categorized into three groups: confirmed, clinically diagnosed, and probable. Confirmed IFD was defined by the identification of fungal elements in sterile specimens through cytology, microscopy, or culture (Dewi et al., 2021). Clinically diagnosed IFD required the presence of host, clinical, and microbiological evidence, such as the detection of fungi or fungal antigens in sterile samples. Probable IFD included patients with compatible host and clinical factors who did not meet the microbiological criteria (Duan et al., 2023).

### mNGS testing and bioinformatic analysis

Pathogen Detection by mNGS: For all ICU patients, BALF samples were collected within 12 hours of admission; Samples from the experimental group were dispatched to the Guangzhou Weiyuan Genomics Company for mNGS analysis. Sample processing involved homogenization using a Bioprep-24 biological sample homogenizer, followed by extraction of total nucleic acids from 300 µL of the supernatant. RNA was reverse transcribed into cDNA, after which a DNA library was constructed. The library underwent purification, amplification, and secondary purification. Sequencing was performed on the Illumina NextSeq CN500 platform with 75 bp paired-end reads. Raw sequencing data were processed with Trimmomatic for quality control. The remaining high-quality microbial reads were aligned to a comprehensive microbiome database developed by Guangzhou Weiyuan Gene Technology Co., Ltd., derived from NCBI RefSeq/NT/NR and augmented with clinical sample data (Gaffney et al., 2023).

In this study, for species-level identification, a positive result was defined as meeting any of the following criteria:

1. The ratio of reads per million (RPM) in the sample compared to the negative control (RPM sample/RPM negative control) is ≥10;
2. The microorganism was ranked among the top 10 in relative abundance in the sample and was not detected in the negative control (NTC).

### Conventional microbiological testing and galactomannan test

Samples were cultured on Sabouraud dextrose agar plates and incubated in a fungal incubator for 2-4 days. The fungal culture was considered positive when the colony count reached ≥10^5^ cfu/mL.

The GM test was conducted using a chemiluminescent immunoassay. A GM concentration ≤0.5 µg/L was interpreted as negative, ≥0.5 µg/L as positive, and the results were interpreted in conjunction with clinical characteristics.

### Assessment criteria and measurement metrics

#### Detection times

The time required to obtain results from mNGS, GM test, and conventional Aspergillus culture was compared.

#### Analysis of pathogen

The distribution of pathogens detected by mNGS and conventional fungal culture was analyzed.

#### Comparison of diagnostic efficacy

The diagnostic performance of mNGS, GM test, conventional Aspergillus culture, and combined testing was compared. The sensitivity, specificity, positive predictive value (PPV), negative predictive value (NPV), and Youden’s Index (J-index) were calculated for each diagnostic modality, including serum GM test, BALF GM test, mNGS, and combined testing strategies.

### Statistical analysis

Statistical analyses were performed using SPSS version 23.0 (IBM Corp., Armonk, NY, USA) and GraphPad Prism version 9.0 (GraphPad Software, San Diego, CA, USA). Normality was assessed using the Shapiro–Wilk test. Data were presented as mean ± SD or median (IQR) and compared using the independent-samples t-test or Mann–Whitney U test, as appropriate. Categorical variables were analyzed using the chi-square or Fisher’s exact test.

Diagnostic performance was evaluated by sensitivity, specificity, PPV, NPV, and Youden’s index. ROC curves were constructed and AUCs compared using the DeLong method, with sensitivity and specificity compared by the McNemar test. A two-sided *P* value < 0.05 was considered statistically significant.

## Results

### Clinical characteristics

**Figure 2:**
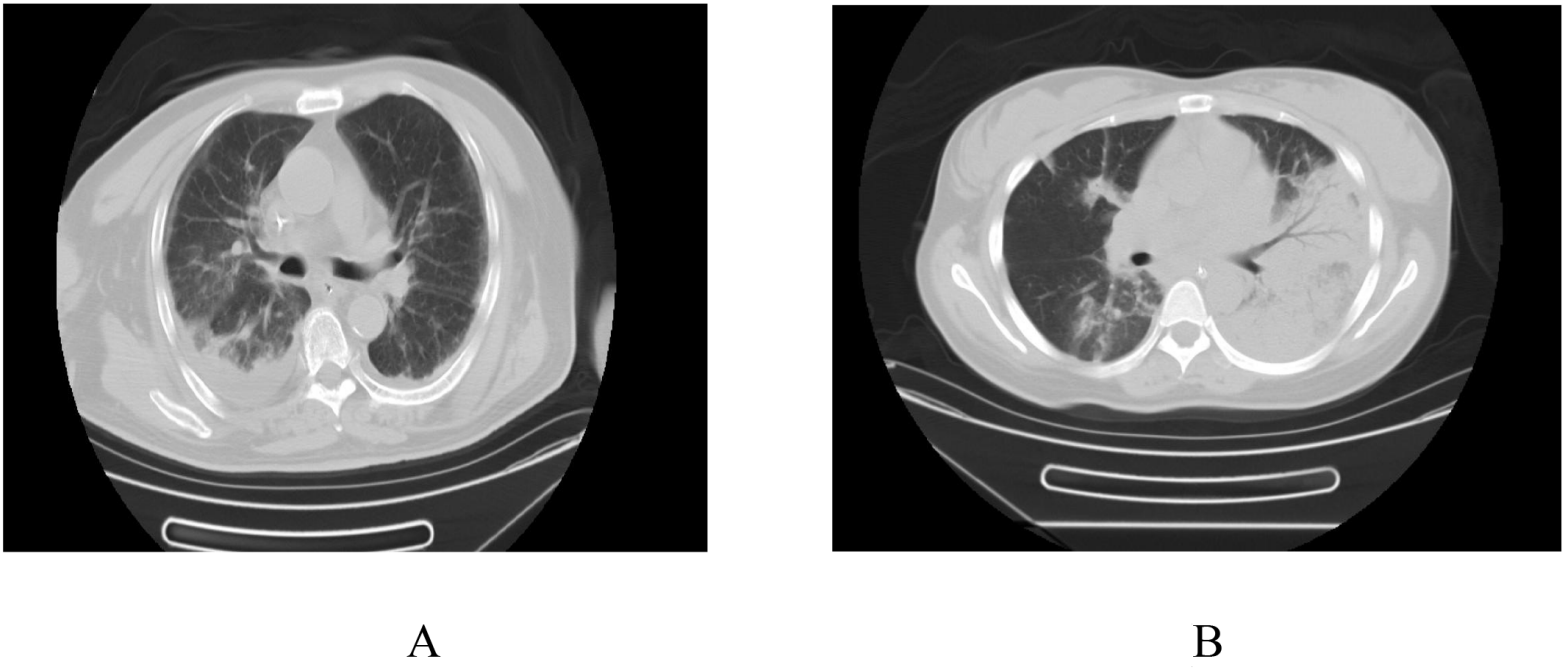
Chest CT findings in patients with influenza complicated by invasive pulmonary aspergillosis: **(A)** Diffuse patchy ground-glass opacities and consolidation are visible in both lungs, with a predominantly interstitial pattern of diffuse infection. **(B)** A large area of consolidation is seen in the left lung, while scattered linear and patchy consolidations are present in the remaining parts of both lungs.

The cohort was stratified into IPA and a non-IPA group. The mean age was comparable between the IPA (68.3 ± 1.1 years) and non-IPA groups (67.9 ± 1.8 years, *P* > 0.05). Clinically, the IPA group exhibited a significantly higher incidence of respiratory distress (56.5% vs. 35.1%, *P* < 0.05) and coma (16.3% vs. 6.7%, *P* < 0.05), whereas rates of cough and sputum production (73.0% vs. 77.2%, *P* > 0.05) and fever (62.3% vs. 65.6%, *P* > 0.05) were similar between groups. Regarding comorbidities, patients in the IPA group had a higher prevalence of diabetes (33.3% vs. 19.8%, *P* < 0.05), COPD (26.9% vs. 14.6%, *P* < 0.05), and liver disease (11.1% vs. 3.1%, *P* < 0.05), whereas the proportion of coronary artery disease was comparable (24.7% vs. 18.8%, *P* > 0.05). Laboratory analyses revealed that the IPA group had a greater proportion of patients with NLR >3 or <0.7 (17.2% vs 6.3%, *P* < 0.05). Moreover, levels of CRP (105.6 ± 42.4 vs. 89.5 ± 36.8, *P* < 0.01), PCT (4.3 ± 3.0 vs. 3.3 ± 2.1, *P* < 0.01), albumin (29.5 ± 3.2 vs. 30.8 ± 4.1, *P* = 0.02), interleukin-6 (91.6 ± 62.4 vs. 70.1 ± 55.4, *P* = 0.01) were significantly higher in the IPA group. Imaging findings demonstrated no statistically significant differences in the prevalence of consolidation (25.5% vs. 20.2%, *P* = 0.28), ground-glass opacities (22.7% vs. 20.7%, *P* = 0.73), or nodular lesions (22.0% vs. 26.7%, *P* = 0.47) between groups. In contrast, cavitary lesions or air crescent signs were more frequent in the IPA group (24.7% vs. 12.4%, *P* = 0.02)(**Figure 2**). Severity scores were higher among IPA patients, with SOFA (9.3 ± 1.9 vs. 8.5 ± 1.3) and APACHE II scores (12.4 ± 4.3 vs. 10.8 ± 2.3) exceeding those of the non-IPA group. The incidence of acute respiratory distress syndrome (ARDS), invasive mechanical ventilation (IMV), vasopressor use, renal replacement therapy (RRT), and mortality was also significantly elevated in the IPA cohort, additionally, the length of ICU stay was longer in the IPA group (14.2 ± 2.3 days vs. 11.6 ± 1.7 days, *P* < 0.01) (**Table 1**).

**Table 1:** Baseline characteristics of the study cohort.

Characteristics	IPA (n=93)	Non-IPA (n=96)	*P* value
Age (years)	68.3 ± 1.1	67.9 ± 1.8	0.78
Sex			
Male	52	47	0.34
Female	41	49	
Symptoms N (%)			
Respiratory distress	52/92 (56.5%)	33/94 (35.1%)	<0.01
coma	14/86 (16.3%)	6/89 (6.7%)	0.04
Cough and sputum production	65/89 (73.0%)	71/92 (77.2%)	0.53
Fever	57/91 (62.3%)	61/93 (65.6%)	0.75
Corticosteroid therapy N (%)			
Cumulative dose 3 m≥ 100mg	23/65 (35.4%)	11/58 (18.9%)	0.04
Comorbidities N (%)			
Diabetes	31/93 (33.3%)	19/96 (19.8%)	0.03
COPD	25/93 (26.9%)	14/96 (14.6%)	0.04
Liver disease	10/93 (11.1%)	3/96 (3.1%)	0.04
Coronary artery disease	23/93 (24.7%)	18/96 (18.8%)	0.32
Laboratory findings at ICU admission			
neutrophil-to-lymphocyte ratios (NLR)>3,<0.7	16/93 (17.2%)	6/96 (6.3%)	0.02
CRP	105.6 ± 42.4	89.5 ± 36.8	<0.01
PCT	4.3 ± 3.0	3.3 ± 2.1	<0.01
Albumin	29.5 ± 3.2	30.8 ± 4.1	0.02
Interleukin-6	91.6 ± 62.4	70.1 ± 55.4	0.01
CD4+ T cell count	289.6 ± 152.9	330.4 ± 189.5	0.03
Imaging findings, N (%)			
Consolidation	23/90 (25.5%)	19/94 (20.2%)	0.28
Ground-glass opacities	20/88 (22.7%)	19/92 (20.7%)	0.73
Cavitary lesions or air crescent signs	21/85 (24.7%)	10/81 (12.4%)	0.02
Nodular lesions	18/82 (22.0%)	23/86 (26.7%)	0.47
Disease severity at ICU admission			
SOFA score, median (IQR)	8.3 ± 1.9	7.1 ± 1.3	< 0.01
APACHE II score	12.4 ± 4.3	10.8 ± 2.3	< 0.01
Acute respiratory distress syndrome N (%)	51/93 (73.2%)	22/96 (22.9%)	< 0.01
Invasive mechanical ventilation N (%)	32/9 (34.4%)	18/96 (18.8%)	0.02
Vasopressors N (%)	23/93 (24.7%)	7/96 (7.3%)	< 0.01
Renal replacement therapy N (%)	10/93 (11.1%)	3/96 (3.1%)	0.04
Alive at ICU N (%)	36/93 (38.8%)	62/96 (64.6%)	< 0.01
Length of ICU stay	14.2 ± 2.3	11.6 ± 1.7	< 0.01
Mortality	30/93 (32.2%)	12/96 (12.5%)	< 0.01

### Comparison of detection times

**Figure 3:**
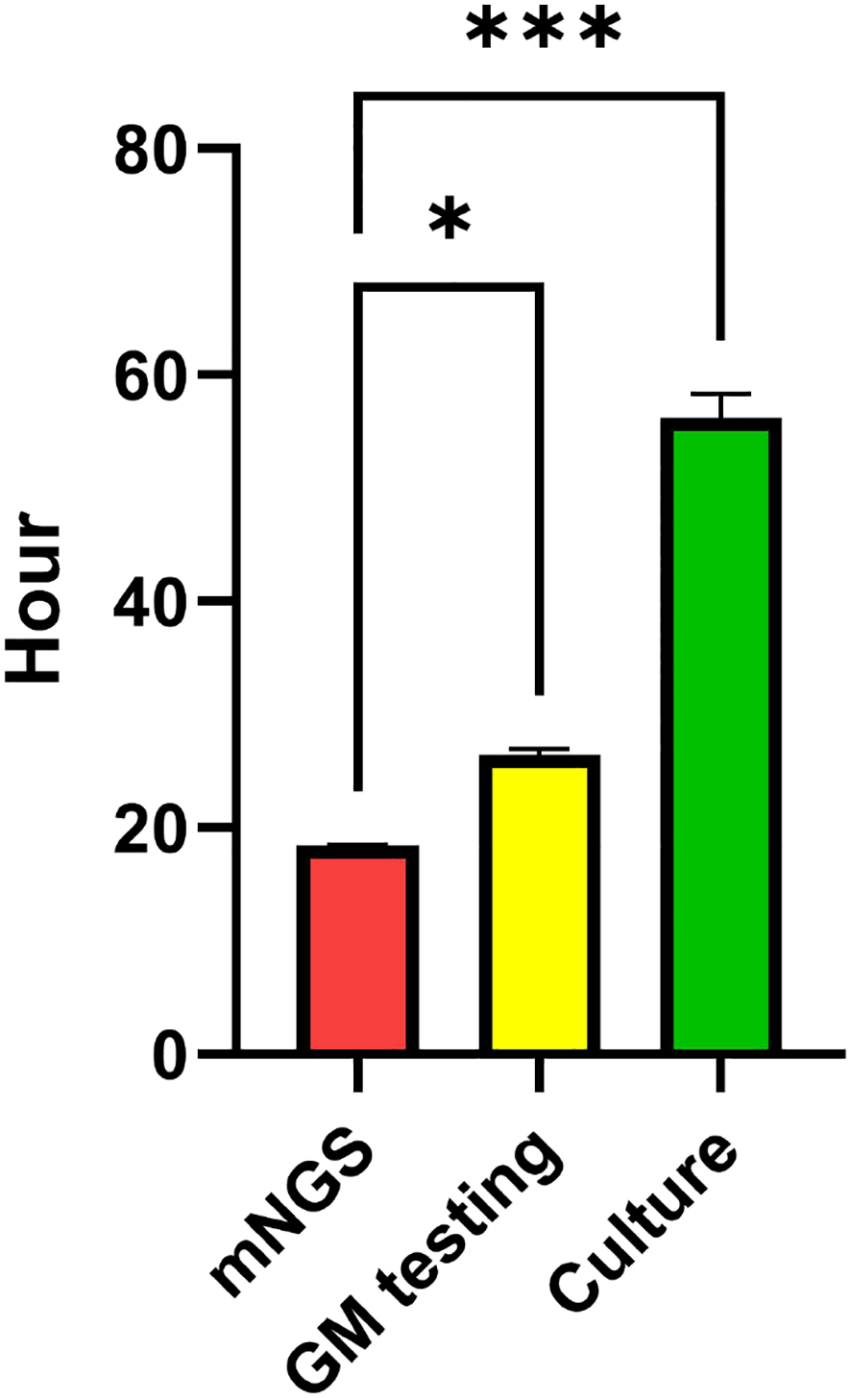
Comparison of detection times among culture, GM testing, and mNGS *P<0.05; *** *P<*0.001.

The time required for mNGS detection (18.8 ± 1.3 hours) was significantly shorter than that for conventional fungal culture (56.7 ± 3.2 hours) and the GM testing (26.1 ± 2.6 hours) (*P* < 0.01) (**Figure 3**).

### Categorization of aspergillus identified by mNGS and conventional culture

**Figure 4:**
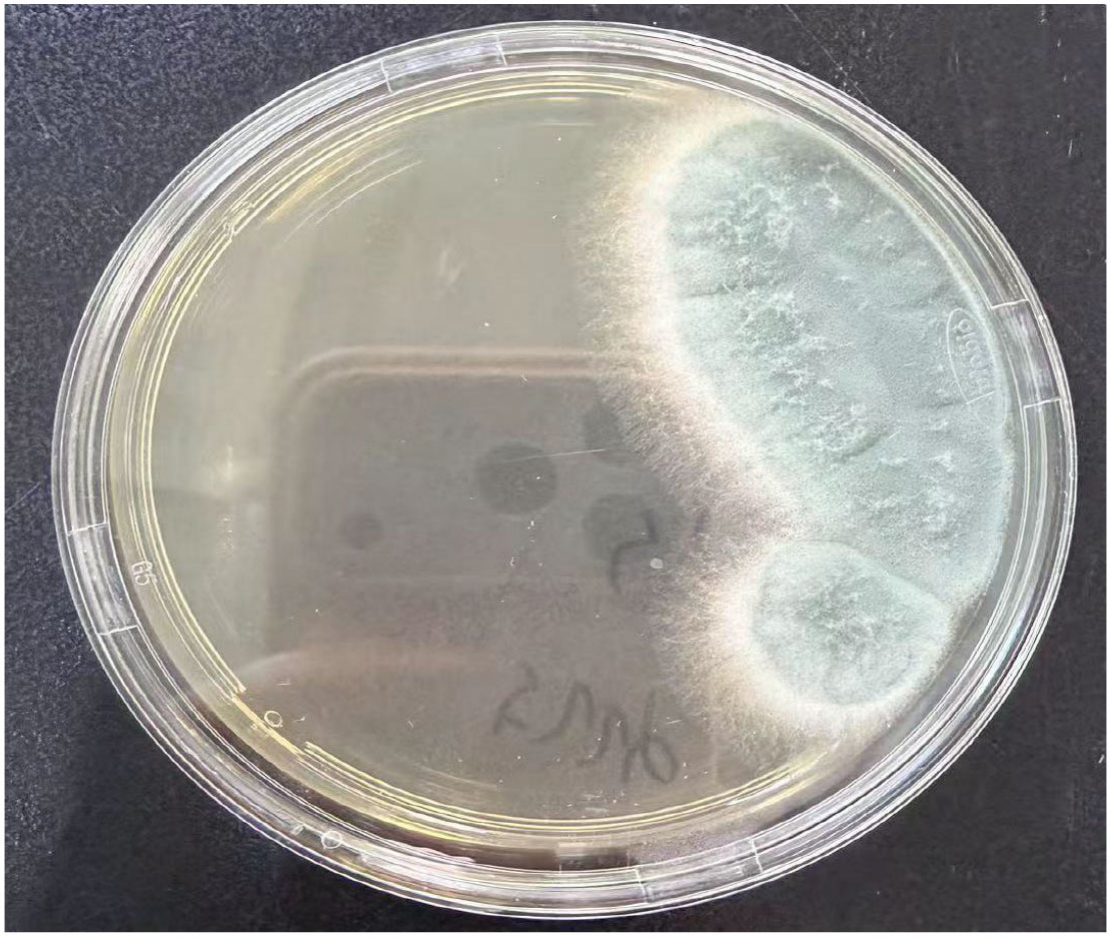
Aspergillus fumigatus.

The mNGS analysis revealed 68 isolates spanning multiple Aspergillus species, while conventional culture methods isolated 27 fungal strains. The distribution of these diverse Aspergillus species is presented in **Table 2**. **Figure 4** presents the results of the conventional fungal culture.

**Table 2:** Spectrum of *Aspergillus* species detected by mNGS and conventional culture.

The species of *Aspergillus*	mNGS	Conventional culture
*Aspergillus fumigatus*	39	18
*Aspergillus flavus*	19	6
*Aspergillus niger*	8	3
*Aspergillus terreus*	2	0

### Comparison of diagnostic performance for severe influenza complicated by IPA using different detection methods

We conducted a comparison of the diagnostic performance of fungal culture, serum GM test, BALF GM test, mNGS, and combined testing methods The sensitivity of mNGS for IPA diagnosis was 72.1%, which was higher than that of traditional fungal culture (28.6%), serum GM test (37.6%), and BALF GM test (44.1%) (*P* < 0.05). When mNGS was combined with GM testing and fungal culture, the sensitivity increased to 81.7%, with a specificity of 91.9%, outperforming any single diagnostic method (**Table 3**). Comparison of sensitivity and specificity across various diagnostic methods for suspected IPA is shown in **Table 4**.

**Table 3:** Comparison of pathogen diagnostic performance among fungal culture, GM test, mNGS, and combined testing.

Method	IPA group (n)	Non-IPA Group (n)	sensitivity(%) (95% CI)	specificity(%) (95% CI)	PPV(%) (95% CI)	NPV(%) (95% CI)	J-index
Culture							
pos	27	10	28.6 (20.2-38.7)	89.5 (81.5-94.3)	73.0 (55.9-85.1)	56.6 (48.5-64.4)	18.1
neg	66	86					
Serum GM							
pos	35	15	37.6 (28.2-48.1)	84.3 (75.8-90.3)	67.3 (53.3-78.8)	57.7 (49.2-65.8)	21.9
neg	58	81					
GM (BALF)							
pos	41	18	44.1 (34.2-54.4)	81.3 (72.3-87.8)	67.2 (54.4-77.9)	59.4 (50.8-67.5)	25.4
neg	52	78					
mNGS							
pos	67	19	72.1 (62.1-80.3)	80.2 (71.1-87.0)	70.5 (60.5-78.9)	72.3 (63.3-79.9)	52.3
neg	26	77					
Combination test							
pos	76	8	81.7 (72.5-88.3)	91.9 (84.4-96.0)	90.4 (81.9-95.3)	83.8 (75.6-89.6)	73.6
neg	17	88					

**Table 4:** Comparison of sensitivity and specificity across various diagnostic methods for suspected IPA, P1 and P2 refer to the comparative analysis of sensitivity and specificity among culture, Serum GM, GM (BALF) and mNGS, respectively.

Culture	*P_2_* = 0.148	*P_2_* = 0.103	*P_2_* = 0.071
*P_1_* = 0.214	Serum GM	*P_2_* = 0.852	*P_2_* = 0.712
*P_1_* = 0.035	*P_1_* = 0.369	GM (BALF)	*P_2_* = 0.855
*P_1_* < 0.01	*P_1_* < 0.01	*P_1_* < 0.01	mNGS

### Comparison of diagnostic performance

**Figure 5:**
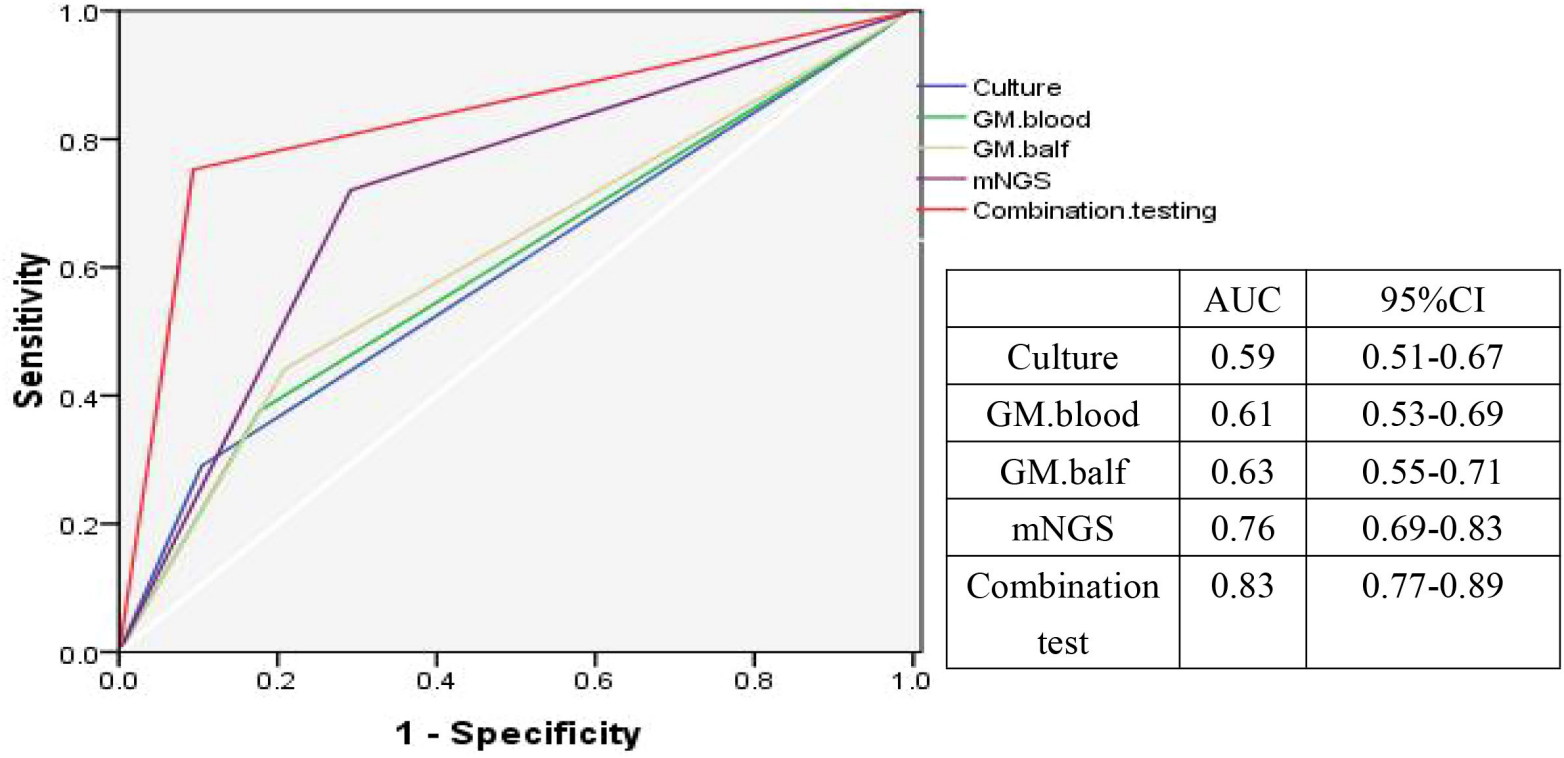
Diagnostic accuracy of different methods as evaluated by ROC analysis.

The AUC for combined testing, mNGS, GM BALF, GM Blood and fungal culture was 0.83, 0.76, 0.63, 0.61, and 0.59, respectively. These results highlight that combined testing provides the most robust diagnostic value for IPA, with mNGS demonstrating superior diagnostic capability over GM and fungal culture alone (**Figure 5**).

## Discussion

Studies on severe influenza complicated by IPA are still relatively limited. This research examined the clinical features, inflammatory profiles, radiological findings, and outcomes of patients with severe influenza complicated by IPA, who were admitted between March 2020 and September 2025. The incidence of IPA in these patients was 49.2%, with a mortality rate of 22.2%(Dewi et al., 2021). To aid in the early and accurate diagnosis of IPA, this study compared the diagnostic performance of fungal culture, GM tests, and mNGS. The results indicate that mNGS provides superior sensitivity and diagnostic efficiency, and that its combination with traditional diagnostic methods further enhances diagnostic performance.

Historically, IPA has been regarded as an opportunistic infection affecting mainly immunocompromised individuals, including those with neutropenia, liver cirrhosis, malignancies, hematopoietic stem cell transplants, or those receiving prolonged corticosteroid or immunosuppressive treatments. However, recent studies have recognized influenza infection itself as an independent risk factor for IPA. In immunocompetent hosts, severe influenza can also result in rapid pulmonary invasion by *Aspergillus* species (Wang et al., 2022).

*Aspergillus* species are ubiquitous environmental fungi. Under normal immune conditions, inhaled Aspergillus conidia are efficiently cleared through mucociliary action and immune phagocytosis (Chen et al., 2020). The innate immune system detects fungal components via pattern recognition receptors and removes them through both reactive oxygen-dependent and -independent mechanisms. Severe influenza predisposes patients to invasive Aspergillus infections through various pathophysiological mechanisms. First, the disruption of respiratory defense barriers. The influenza virus damages airway epithelial cells, impairs mucociliary clearance, and disrupts the alveolar-capillary barrier, providing a favorable niche for fungal colonization and invasion (Boyd and Martin-Loeches, 2021).Second, virus-induced immunosuppression occurs. Influenza infection impairs the phagocytic function of alveolar macrophages and reduces circulating lymphocytes, including CD4+ T cells, resulting in compromised cellular immunity. This immunosuppressive state, sometimes referred to as “immune paralysis,” allows Aspergillus to escape immune surveillance and proliferate within the host (Niu and Zhao, 2022). Third, secondary susceptibility due to medical interventions, such as broad-spectrum antibiotics and corticosteroids.

Since the initial report by J.J. Fischer, who described two cases of influenza A infection complicated by IPA, the number of documented severe influenza cases associated with IPA has rapidly and consistently increased (Fischer and Walker, 1979).A study in The Lancet Respiratory Medicine found an IPA incidence of 19% in patients with severe influenza, with a mortality rate of 51% (Schauwvlieghe et al., 2018). In this cohort, the majority of affected individuals were male, and many had a history of prolonged corticosteroid exposure. Moreover, the in-hospital mortality rate was significantly higher among patients with IPA compared to those without (49.2% vs. 27.0%), which is consistent with the results of this study (Lee et al., 2024). Another study demonstrated that the median interval between influenza diagnosis and antifungal treatment was two days in surviving IPA patients, compared to nine days in non-survivors, highlighting the importance of early recognition and intervention (Qian et al., 2020). Therefore, for patients with a strong clinical suspicion of IPA, empirical antifungal therapy should be initiated promptly during diagnostic evaluation.

Although IPA is clinically significant, its symptoms are often nonspecific, Traditional diagnostic methods for *Aspergillu*s infection include fungal culture, G test, and GM detection. Although histopathology and tissue culture are considered the gold standards, they are often impractical in critically ill influenza patients with acute respiratory distress or hemodynamic instability due to the risks associated with the procedures. Moreover, microbial culture relies on the growth of viable organisms, with fungal culture typically requiring 2-4 days for definitive results. In contrast, mNGS can identify pathogens within 24 hours, significantly reducing diagnostic turnaround time. The GM test, which detects the *Aspergillus* cell wall polysaccharide antigen via enzyme-linked immunosorbent assay (ELISA), is widely used for IPA diagnosis. Multiple studies have demonstrated that BALF GM testing offers superior sensitivity compared with serum GM testing. This increased sensitivity is attributed to the predominance of airway invasion over vascular invasion in influenza-associated IPA and the ability of neutrophils in immunocompetent hosts to restrict Aspergillus dissemination into the bloodstream, thus lowering the diagnostic sensitivity of serum GM assays.

As the management of IPA is highly time-sensitive, mNGS, as a high-throughput sequencing technology, enables rapid and comprehensive identification of all microorganisms, including bacteria, viruses, and fungi, without requiring prior knowledge of the causative pathogens. This capability provides mNGS with a distinct advantage in diagnosing *Aspergillus* infection (Niu et al., 2024; Feys et al., 2024). Research has demonstrated that, among neutropenic patients, the sensitivity of BALF mNGS for diagnosing IPA can reach 92.31%, exceeding that of traditional diagnostic methods and GM testing. Furthermore, the AUC for mNGS in diagnosing *Aspergillus* infection has been reported at 84.7%, significantly higher than that of the GM test (72.7%), underscoring the superior diagnostic accuracy of mNGS.

This study found that the sensitivity of BALF mNGS was 72.1%, outperforming BALF GM (44.1%), serum GM (37.6%), and conventional fungal culture (28.6%). BALF mNGS also exhibited a shorter detection time. However, its specificity was lower than that of conventional fungal culture. Notably, combined testing yielded a sensitivity of 81.7% and specificity of 91.9%. The AUC for BALF mNGS was 0.76, significantly higher than those for BALF GM (0.63), GM (0.61), and conventional fungal culture (0.59), while the combined diagnostic approach achieved an AUC of 0.83, aligning with previous reports and indicating improved diagnostic efficacy for IPA.

Distinguishing between *Aspergillus* colonization and invasive infection in lower respiratory tract specimens requires comprehensive evaluation of host risk factors, clinical manifestations, radiologic findings, and mycological evidence. In patients with significant predisposing factors—namely corticosteroid exposure, immunosuppression, or severe influenza—who develop new or worsening respiratory symptoms accompanied by radiologic progression and positive mycological assays (culture, galactomannan, or mNGS), this constellation of findings is highly suggestive of invasive pulmonary aspergillosis. Conversely, isolation of Aspergillus in the absence of clinical or radiologic deterioration favors colonization rather than invasive disease.

Despite these promising results, this study also has several limitations. Primarily, the research was a retrospective analysis conducted at a single institution with a small sample size, which limits the generalizability of the findings. Additionally, the number of clinically diagnosed IPA cases exceeded that of confirmed IPA cases, potentially introducing diagnostic bias. Finally, the final diagnosis often involves a degree of subjective clinical judgment in distinguishing colonization from true infection, requiring integration of patient history, radiological findings, and laboratory results. Therefore, prospective multicenter studies with larger cohorts are warranted to further validate the diagnostic utility of mNGS for severe influenza complicated by IPA.

## Conclusion

The incidence of invasive *Aspergillus* infection in patients with severe influenza has been increasing, contributing to high mortality and poor clinical outcomes. Timely and accurate diagnosis, followed by appropriate treatment, is crucial for improving patient outcomes. mNGS offers a rapid, sensitive, and comprehensive diagnostic methodology, with substantial potential to facilitate the early diagnosis of IPA, improve diagnostic accuracy, minimize misdiagnoses, and enable the prompt adjustment of treatment strategies.

## Data Availability

The original contributions presented in the study are included in the article/supplementary material. Further inquiries can be directed to the corresponding author.
